# Potentially large post-1505 AD earthquakes in western Nepal revealed by a lake sediment record

**DOI:** 10.1038/s41467-019-10093-4

**Published:** 2019-05-21

**Authors:** Z. Ghazoui, S. Bertrand, K. Vanneste, Y. Yokoyama, J. Nomade, A. P. Gajurel, P. A. van der Beek

**Affiliations:** 1 ISTerre, Institut des Sciences de la Terre, Université Grenoble Alpes, CNRS, 38058 Grenoble, France; 2 RCMG, Renard Centre of Marine Geology, Ghent University, 9000 Ghent, Belgium; 3 Department of Seismology and Gravimetry, Royal Observatory of Belgium, 1180 Brussels, Belgium; 4 Atmosphere and Ocean Research Institute, The University of Tokyo, 277-0882 Chiba, Japan; 5 Department of Geology, Tribhuvan University, 44618 Kathmandu, Nepal

**Keywords:** Sedimentology, Geodynamics

## Abstract

According to paleoseismological studies, the last earthquake that ruptured the Main Frontal Thrust in western Nepal occurred in 1505 AD. No evidence of large earthquakes has been documented since, giving rise to the concept of a seismic gap in the central Himalaya. Here, we report on a new record of earthquake-triggered turbidites from Lake Rara, western Nepal. Our lake-sediment record contains eight possibly moderate-to-large earthquake-triggered turbidites during the last 800 years, three of which overlap in age with previously reported *M*_w_ ≥ 7 events in western Nepal. Shaking intensity modelling, together with instrumental records, suggests that near-field earthquakes (≤15 km) should have a minimum *M*_w_  5.6, and regional earthquakes (≤80 km) a *M*_w_ > ~ 6.5, to trigger turbidites. We present a likely scenario that western Nepal may be as seismically active as central Nepal; however, more data are needed to revaluate the seismic risk in the central Himalaya.

## Abstract (web summary)

Sediments have the potential to preserve the signature of geologic events such as earthquakes. Here, the authors provide a paleoseismological analysis of the sediments of Lake Rara, Nepal, to reconstruct the number of earthquakes that caused lake shaking and subsequent turbidite deposition during the last centuries.

## Introduction

The Himalayan collision, in which India underthrusts below Tibet and the Himalaya along a major crustal detachment known as the Main Himalayan Thrust (MHT), regularly produces major destructive earthquakes, as elastic deformation accumulated during underthrusting of the Indian plate is released periodically by slip along the MHT fault plane^1–4^. The destructive 2015 *M*_w_ 7.8 Gorkha earthquake^5,6^ represented an intermediate-size event in this process as it ruptured only the lower, northern part of the MHT without breaking through to the surface^4,5^.

**Fig. 1:**
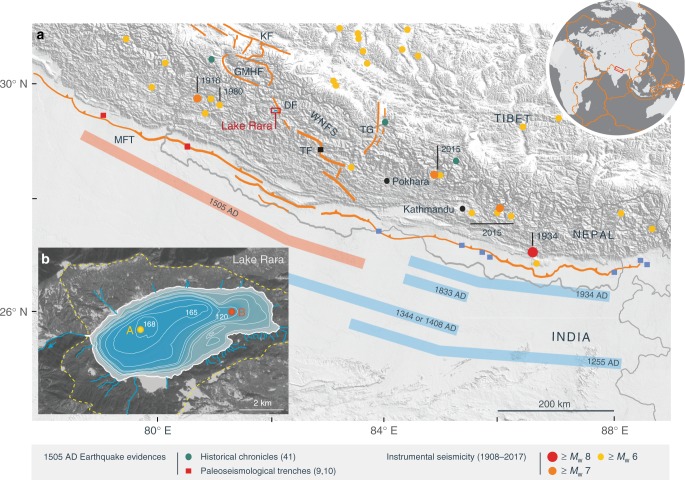
Geomorphic and seismotectonic setting of Lake Rara. **a** ASTER Global Digital Elevation model (GDEM) of the central Himalaya, showing active faults^37,57^, instrumental seismicity (*M*_w_ ≥ 6) from 1908–2017 (2017 update of ISC-GEM^34^), and inferred rupture lengths of historical earthquakes (dates as indicated). Squares indicate paleoseismological trenching sites, in red for the great 1505 AD earthquake, in black for the Tibrikot earthquake and in blue for central Nepal earthquakes (see main text for references). Green dots are locations for which historical chronicles record destruction during the 1505 AD earthquake^58^. Instrumental seismicity is coloured according to magnitude, earthquakes discussed in the text are indicated by date. Active faults are: KF—Karakoram Fault; MFT—Main Frontal Thrust; TG—Thakkola Graben; WNFS—Western Nepal Fault System (composed in part by: DF—Dharma Fault; GMHF—Gurla Mandhata-Humla Fault system; TF—Tibrikot Fault). The red rectangle shows the location of Lake Rara (inset **b**). **b** Bathymetric map of Lake Rara (from ref. ^40^ and our own measurements) showing the sampling sites A and B, overlain on a satellite image of the lake catchment (outlined by yellow dashed line, the full catchment is shown in Supplementary Fig. 1; Landsat-7 image courtesy of the U.S. Geological Survey). ASTER GDEM is a product of NASA and METI

Evaluation of seismic hazard in the Himalaya has been based on the comparison of geodetic strain rates with the amount of seismic moment release, measured by instrumental seismicity and inferred from paleoseismology^1,7^. Whereas historical seismicity and trenching studies record at least four, and possibly up to eight, major earthquakes over the last 800 years in central and eastern Nepal^8^, the last known major event to have affected western Nepal and northern India, rupturing a long portion of the Main Frontal Thrust (MFT), was the *M*_s_ ~8.2 earthquake of 1505 AD^9,10^ (Fig. 1). The intervening 500 years have resulted in the accumulation of a >10 m slip deficit along this segment of the MHT, leading to the concept of a seismic gap in western Nepal and adjacent areas in northern India, which could potentially trigger a great earthquake in the near future^1,7,11^.

However, both historical and paleoseismic records of earthquakes are inherently incomplete^8,12,13^ and the age, extent, and correlation of surface ruptures inferred from paleoseismic trenching studies in the central Himalaya are the subject of significant controversy^6,8,14–17^.

Historical written archives in western Nepal are limited due to several factors. For the period prior to the 20^th^ century, references to earthquakes in western Nepal and the northern Himalaya are scattered throughout Tibetan literature and, until the mid-20^th^ century, access to Tibet and Tibetan documents was extremely restricted to foreign researchers^12^. Moreover, translation and interpretation of traditional references to earthquakes is complicated by the religious undertone in the Tibetan hagiographic literature^12^. In addition, western Nepal has always been much less densely populated than central Nepal, lacking major urban centres (such as Kathmandu, Pokhara or Gorkha) and it was virtually inaccessible to foreign researchers during the Nepal civil war of 1996–2006. Furthermore, seismic activity based on written archives is exclusively evaluated in terms of macroseismic effects and is subject to misinterpretations^12^, as the vulnerability of buildings exposed to earthquakes varies greatly in the Himalayan region depending on the type of architecture. In particular, traditional timber-laced buildings common to the western Himalaya (including western but not central Nepal)^18,19^ have proven their ability to resist earthquake loading much better than modern structures, but their exceptional resistance is not taken into account in any of the intensity scales^12,18,19^.

Trench-based paleoseismic records are rendered equivocal by the strong vegetation cover and erosional activity of the Himalayan front, leading to poor access and preservation potential of fault scarps. Furthermore, the unknown and variable age inheritance in the charcoals used for radiocarbon dating of observed surface ruptures, i.e., the time lapse between charcoal formation and its incorporation in the sediments from which it is sampled^20^, may lead to significant overestimation of the ages of seismic events^8^.

Finally, trenches on the Himalayan front only record surface-breaking earthquakes. However, not all earthquakes produce slip that reaches the surface (e.g., the 2015 *M*_w_ 7.8 Gorkha earthquake^5,6^) and, therefore, would be detected from trenches. Moreover, while some ruptures reach the surface at the MFT, producing fault escarpments or fault-related folds, others (e.g., the 2005 *M*_w_ 7.6 Kashmir earthquake)^4^ reach the surface through out-of-sequence thrusting.

Compared to the historical and terrestrial archives discussed above, lake sediments may provide a complementary and more continuous paleoseismic record, as earthquake-triggered slope failures and surficial sediment remobilization form turbidite deposits^21–27^. In spite of their potential to complement the paleoseismic inventory, lake records have hitherto not been investigated in the Himalaya.

**Fig. 2:**
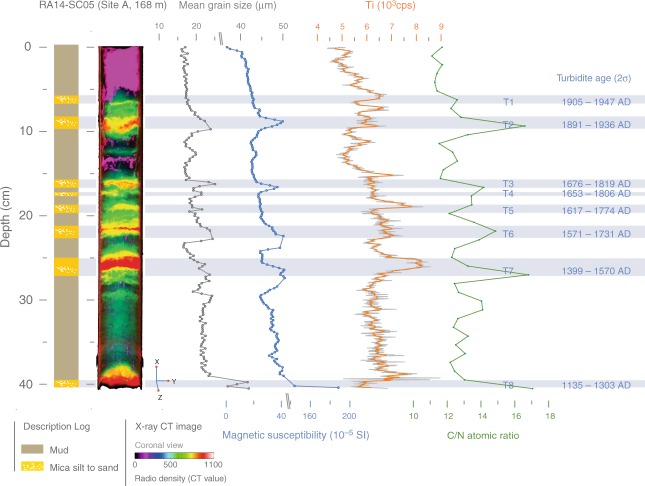
Identification of turbidites in sediment core RA14-SC05. From left to right are shown: a synthetic log, 3D coronal view of computed X-ray tomography images in pseudo-colour, mean grain size, magnetic susceptibility, Itrax XRF Ti profile (grey curve: raw data; orange curve: 5 pt-weighted average) and carbon to nitrogen (C/N) atomic ratio. These five parameters were used to identify turbidites T1 to T8. Their ages were extracted from the age-depth model (see Methods)

In order to reconstruct past earthquake activity in the inferred seismic gap, we collected three short sediment cores from Lake Rara in western Nepal (29°32’N, 82°05’E; Fig. 1; Supplementary Fig. 1). We cored two sites within the lake, selected on the basis of a bathymetry survey (See Methods), in the deepest basin at water depths of 168 m (site A) and in the shallower northeastern arm of the main basin at 120 m depth (site B), using a gravity corer operated from an inflatable dingy (Fig. 1). The cores were analysed using X-ray Computerized Tomography (CT), X-ray fluorescence (XRF) core scanning, logging of physical properties, bulk organic geochemistry, and high-resolution grain-size measurements (Fig. 2; Supplementary Figs. 2, 3; see Methods). Chronology was established on cores RA14-SC05 and RA14-SC06 by combining radionuclide (^210^Pb and ^137^Cs) and radiocarbon (^14^C) dating (Supplementary Fig. 4, Supplementary Tables 1–3). Radiocarbon dating was performed on terrestrial leaf material and thus does not suffer from age inheritance. Our lake-sediment record contains eight earthquake-triggered turbidites during the last 800 years, five of which were previously undocumented and may represent large seismic events. These results suggest that western Nepal may be as seismically active as central Nepal and call for a revaluation of the risk of a major earthquake affecting western Nepal and northern India.

## Results

### Lake Rara turbidites

The cored sediments are mainly composed of mud, interrupted by 10–25 mm thick, dense, magnetic and Ti-rich micaceous sandy silt layers (Fig. 2), which are characterized and differentiated on the basis of variations in mean grain size, magnetic susceptibility, Ti concentration, C/N ratios and radio-density (see Methods). As their distinctive signatures on the magnetic susceptibility and XRF profiles clearly contrast with the homogeneous muddy background, these fining-upward deposits were readily identified as turbidites (see Supplementary text). Up to eight of these deposits were identified in the cores; they are most clearly represented in core RA14-SC05 (Fig. 2; Supplementary Fig. 2). Age-depth models (Supplementary Fig. 4; Methods) show that sedimentation rates are ~0.4 mm/yr at site A, located in the deepest part of the lake (168 m), and approximately half that at site B. Both the lake physiography and the sedimentation rates at site A favour the ability of this site to record a complete event history^23,24^; we, therefore, focus here on the interpretation of core RA14-SC05.

### Origin of turbidites

Turbidites within lake sediments can be triggered by various processes such as floods, landslides, spontaneous slope failures, or earthquakes (see Supplementary text). The limited hydrographic system (Fig. 1, Supplementary Fig. 1), with short (the largest stream length is ~4 km from source to lake) and small (streams entering the lake are typically less than 30 cm wide and 15 cm deep) streams, and the relatively low relief of the Lake Rara catchment, render floods and landslides unlikely triggering mechanisms. This inference is consistent with the very low sedimentation rates measured at the shallowest site RA14-SC06 (~0.2 mm/yr), which reflects the low hydrodynamic activity in the catchment of Lake Rara, and render spontaneous slope failures also unlikely. Hence, the turbidites identified in the sediments of Lake Rara most likely represent either earthquake-triggered slope failures or earthquake-triggered remobilization of surficial lake sediments. This interpretation is supported by their C/N ratios, which range between 13 and 17 (Fig. 2), reflecting a mixture of organic matter of aquatic (C/N < 8) and terrestrial (C/N > 20) origin^22^. Although the absolute values depend on grain size (Supplementary Fig. 3), these results suggest that the turbidites originated from reworking of sediment previously deposited on shallower parts of the slopes within the lake, where C/N values are slightly higher than in the centre of the lake due to continuous input of terrestrial organic matter from the catchment. Flood or landslide-triggered turbidites, in contrast, would have a purely terrestrial C/N signature^22^.

The strongest argument for attributing an earthquake origin to lake turbidites is a temporal correlation with known historical events^22,23,26^. A good starting point for this correlation is the great 1505 AD earthquake. Historical records describe widespread devastation in western Nepal, northern India and southern Tibet (Fig. 1), from which a magnitude *M*_s_ 8.2 has been estimated^12^. Surface ruptures on the MFT immediately south and southwest of Lake Rara have been attributed to the 1505 event^9,10^. This earthquake, therefore, seems an obvious candidate to have triggered slope failures within Lake Rara. The age range of turbidite T7 in core RA14-SC05 (1399–1570 AD) overlaps the timing of the 1505 AD earthquake. This is the most prominent event deposit; it can be correlated to turbidite TA in core RA14-SC06 from site B based on the independent age models (Supplementary Figs. 4, 5), and it is present in all three cores (Supplementary Fig. 2). We suggest that these events have been caused by the great 1505 AD earthquake.

Another known earthquake that affected the region of Lake Rara is the 1916 *M*_w_ ~7.0–7.2 Dharchula earthquake^28^. The ages of the two topmost turbidites in our record (T1 and T2; Fig. 2; Supplementary Fig. 4) both overlap with this earthquake, with the weighted-mean age of T2 (1917) being very close to it. Therefore, it appears likely that the 1916 earthquake triggered either T1 or T2, but the age overlap between these does not allow us to pick one or the other (Supplementary Fig. 4). The association of these two turbidites with known earthquakes supports the seismic nature of the lake turbidites recorded in Lake Rara. The others (Fig. 2) thus most likely represent previously undocumented earthquakes.

### Earthquake turbidite-triggering threshold

**Fig. 3:**
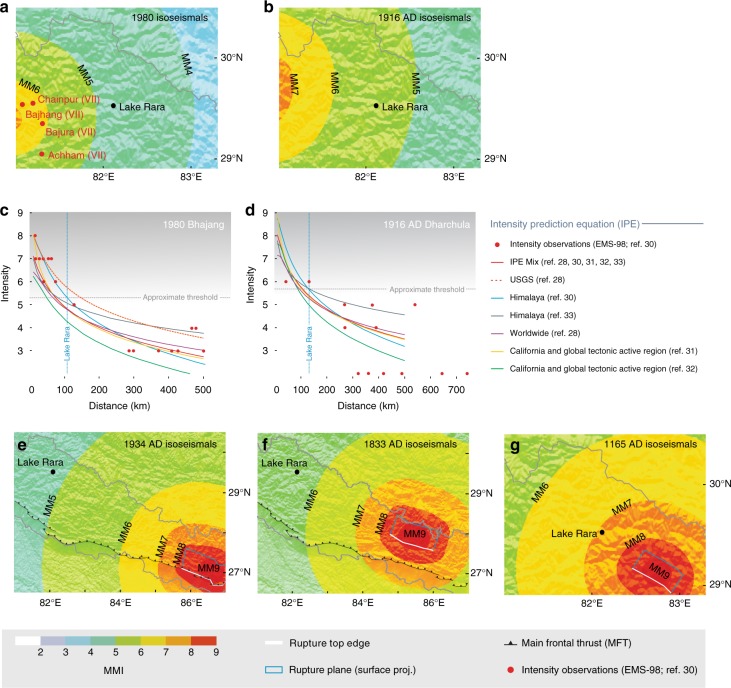
Modified Mercalli Intensity isoseismals modelled for historical and instrumental earthquakes in central/western Nepal, and plots of observed and predicted intensity vs. distance. Modelled isoseismals and observed intensities for instrumental earthquakes in western Nepal; **a** 1980 (MHT?) Bhajang earthquake (modelled *M*_w_ 6.5; intensity observations^28^ in map region are indicated), **b** 1916 (MHT?) Dharchula earthquake (modelled *M*_w_ 7.2; no intensity observations in map region). **c**, **d** Plots of observed and predicted intensity vs. distance for the 1980 Bhajang (**c**) and 1916 Dharchula (**d**) earthquakes, respectively. The vertical blue line corresponds to the epicentral distance of Lake Rara; the dashed grey lines indicate the lower (**c**) and upper (**d**) bounds of our approximate local earthquake turbidite-triggering threshold. Red dots are observed intensities^28^, coloured lines show predicted intensity vs. distance according to the different IPEs^28,29,31–33^ and their average. Also shown is USGS Shakemap fit for the 1980 Bhajang earthquake (see Methods for discussion). **e**–**g** Modelled isoseismals for historical earthquakes in central/western Nepal; **e** 1934 MHT earthquake (modelled *M*_w_ 8.4), **f** 1833 MHT earthquake (modelled *M*_w_ 7.7) and **e** 1165–1400 AD Tibrikot Fault (part of the WNFS) earthquake (modelled *M*_w_ 7.9) The surface projection of the rupture plane is represented by the grey rectangle; the top edge of the rupture is shown by a solid white line. Rupture scenarios for **e** and **f** are from ref. ^35^; for **g** from ref. ^37^. The location of Lake Rara is indicated

Observations in lakes worldwide suggest that earthquake-triggered turbidites can originate either from subaqueous slope failures or from surficial sediment remobilisation, when local shaking exceeds Modified Mercalli Intensities (MMI) 6–7 and 6, respectively^21–24,26,27^. In order to predict shaking intensity and constrain the local earthquake turbidite-triggering threshold (E_Q_TT)^24^ for Lake Rara, we produced MMI shaking maps for several historical and instrumental events (Fig. 3) based on a set of intensity-prediction equations^28–33^ (IPEs; see Methods).

The E_Q_TT for Lake Rara was estimated using two instrumental earthquakes; the 1980 *M*_w_ 6.5 Bajhang and 1916 *M*_w_ ~7.0–7.2 Dharchula earthquakes. The most recent update of the ISC-GEM catalogue^34^ reassessed the location and magnitude of the Dharchula earthquake at an epicentral distance of ~130 km from Lake Rara (29.730°N 80.745°E; Supplementary Fig. 8). As discussed above, this earthquake can be correlated to T1 or T2 in the sediments of Lake Rara. The 1980 *M*_w_ 6.5 Bajhang earthquake^34^ (80-km epicentral distance from Lake Rara) constitutes the largest among six *M*_w_ 6.2–6.5 instrumental earthquakes recorded in the 1974–2017 USGS catalogue within an 80-km radius of Lake Rara (Supplementary Fig. 8). Since their ages are much younger than the most recent turbidite T1 in Lake Rara (Fig. 2), we infer that those six *M*_w_ 6.2–6.5 earthquakes did not trigger slope failures or surficial sediment remobilization in the lake. The E_Q_TT for Lake Rara should, therefore, lie between the shaking intensities felt at the lake for these two large earthquakes. Mean shaking intensities obtained with our IPE selection suggest that the E_Q_TT is situated between MMI ~4.5 and MMI ~5.5 (Fig. 3a, b). However, comparison with observed intensities for the Bajhang earthquake^28^ suggests that our modelled near-field intensities may underestimate actual intensities by a half to one unit (see Methods; Fig. 3c, d). From the IPEs in our selection, it appears that the IPE calibrated for India and the Himalaya^28^ shows the best match with the observed intensities^28^ in the distance range of interest to this study. Hence, with the available data and the associated uncertainties, we cannot tightly constrain the E_Q_TT for Lake Rara, although a value close to the lower bound of the range reported in the literature (MMI 6) is likely. In the following, we will, therefore,use an indicative estimate of the local E_Q_TT in the range of MMI 5.3–5.7, based on the Himalayan IPE^28^.

### Possible correlation with other historical earthquakes

Attributing T1 or T2 to the *M*_w_ ~7.0–7.2 1916 Dharchula earthquake and turbidite T7 to the 1505 *M*_s_ ~8.2 earthquake implies that five other post-1505 AD events are recorded in Lake Rara sediments (Fig. 2), some of which may correspond to known earthquakes that occurred in Nepal or northern India during the last two centuries. In particular, the 1833/08/26 and 1934/01/15 earthquakes affecting central and eastern Nepal (Supplementary Fig. 6) are well documented both geologically and macroseismically (Supplementary Table 5). Both earthquakes ruptured the MHT east of Kathmandu at epicentral distances of ~365 km and ~545 km from Lake Rara, respectively, and have been attributed moment magnitudes *M*_w_ ~7.3–7.7 and ~8.1–8.4, respectively^8,28,30^. Surface ruptures on the MFT were attributed to the latter event^8,13,15,30^, although this interpretation has recently been questioned^17^. Published isoseismals for these earthquakes vary significantly but in most of these, Lake Rara lies outside the MMI = 6 isoseismal^15,28,30^. We have modelled the isoseimals for both earthquakes using the rupture planes and magnitudes inferred by ref. ^35^ (Fig. 3, Supplementary Fig. 6). For the 1934 earthquake, modelled intensities at the lake are MMI < 5, whereas they are 5 < MMI < 6 for the 1833 earthquake. For this far-field earthquake, our predicted intensities correspond much better to the observed intensities than for the near-field earthquakes discussed above. We thus conclude that these modelled intensities are reasonable and that the 1934 event is not likely to have generated a turbidite in the lake, even though it is consistent with the age of turbidite T1. Either T1 or T2 thus corresponds to a previously unknown late 19^th^–early 20^th^-century event, which could have gone unnoticed if it was not felt in India. Whereas modern documentation of earthquakes (including instrumental seismicity, newspaper reports, geological accounts) goes back to this time in India, such documentation for western Nepal only exists since the 1970’s. The 1833 event could possibly have triggered a turbidite in lake Rara, but none of the turbidites overlaps with this age. Likewise, the 1803/09/01 Kumaon earthquake, at an epicentral distance of ~385 km, has been attributed a magnitude *M*_w_ ~7.3–7.5^28,30^. Published isoseismals (Supplementary Fig. 7) suggest an intensity of IV (MSK) for Lake Rara^30^, which is clearly insufficient to trigger a turbidite. We did not attempt to model isoseismals for this earthquake, given the expected low intensities at Lake Rara.

The Lake Rara sediment record also includes at least one medieval earthquake (T8 in core RA14-SC05; Fig. 2, Supplementary Fig. 4). Although two or three great medieval earthquakes were inferred from paleoseismic trenches in Nepal, of which the historic 1255 earthquake has attracted the most attention, their exact magnitude and extent of slip remain controversial^8,14–16^. Surface ruptures attributed to the 1255 earthquake have recently been documented from locations to the south and southwest of Kathmandu^36^. However, given the apparent similarity of the 1255 and 1934 events^8,35^, we consider it unlikely that T8 was triggered by the 1255 AD earthquake.

The Western Nepal Fault System (WNFS) has been recognised as an active trans-tensional fault system that accommodates oblique convergence in the western central Himalaya and connects to the Karakorum Fault^37,38^ (Fig. 1). Although the activity of this system cannot be resolved from geodetic data^39^, it presents features that are diagnostic of active faulting. In particular, a surface-rupturing event has been documented on the Tibrikot Fault (Fig. 1), tentatively dated between 1165 and 1400 AD and attributed to an *M*_w_ ~7.9 earthquake^37^. Our modelling shows that Lake Rara is within the MMI = 7 isoseismal for this rupture scenario (Fig. 3e). We, therefore, suggest that turbidite T8 (1135–1303 AD) records seismic shaking associated with the Tibrikot earthquake^37^.

### Significance of previously unknown events

**Fig. 4:**
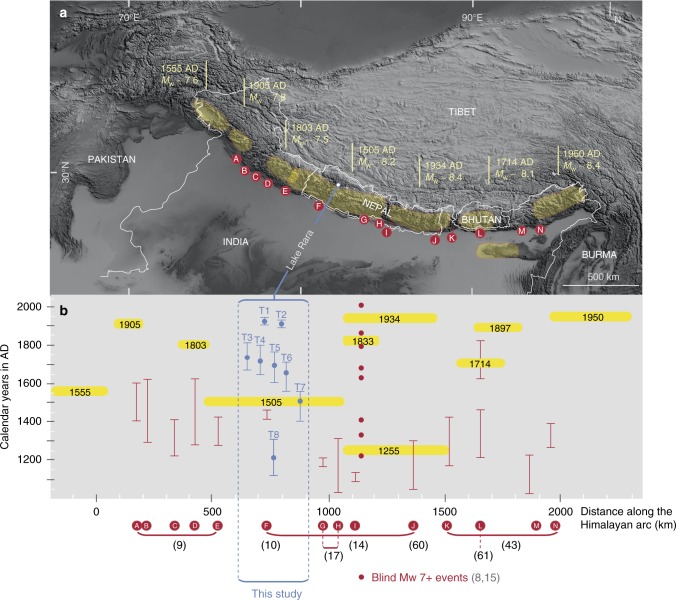
Overview of historical and paleoseismic data along the Himalayan front. The map is modified and updated from ref. ^59^. **a** Digital terrain model of the Himalayan region. Yellow areas correspond to inferred ruptures of historical earthquakes (the surface ruptures of the 1255 AD and 1833 AD earthquakes overlap that of the 1934 earthquake and are not represented); their timing and magnitude are indicated. Red dots labelled A to N represent paleoseismic trenches along the MFT; references are given in parentheses below plot (**b**). **b** Ages and extents of historical Himalayan *M*_w_  >  ∼7.5 earthquakes (horizontal yellow bars). Red vertical bars reflect ^14^C ages that bracket the age of surface ruptures in calendar years AD, with 2σ uncertainties. Blue vertical bars represent the turbidite ages extracted from the age-depth model (2σ; this study); blue dots indicate the weighted mean age. Data from refs. ^8–10,14,15,17,43,60,61^

Overall, five turbidites post-dating the great 1505 earthquake cannot be attributed to any previously described earthquake (Fig. 4). To evaluate whether or not background seismicity (*M*_w_ < 5.6) could have triggered these turbidites, we consider all earthquakes reported by the USGS within a radius of 20 km of Lake Rara between 1974 and 2017, the period during which the USGS catalogue is considered complete. Our search yielded 11 earthquakes with *M*_w_ ranging from 4 to 5.6, none of which is recorded as a turbidite in Lake Rara. These results suggest that background seismicity, i.e., events *M*_w_ < ~ 6, do not significantly remobilize surficial sediments to generate turbidites in Lake Rara. In addition, we tested the sensitivity of the lake site to near-field moderate to large earthquakes. In this test (see Methods), we used the aforementioned IPEs to infer the magnitude required to reach the inferred approximate (MMI 5.3–5.7) threshold intensity at Lake Rara for hypothetical mid- to upper-crustal moderate-magnitude events on the closest known faults (Supplementary Fig. 8): one on the MHT directly below the lake (at a depth of 26 km^39^), and two others on the Dharma (DF) and Hula (HF) Fault segments of the WNFS, at epicentral distances of <15 km and 80 km, respectively. The results (Supplementary Fig. 8) indicate that, in order to be recorded in Lake Rara sediments, nearby earthquakes should have a minimum magnitude *M*_w_ ≥ 5.3–5.6 for the Rara-MHT and DF scenarios, with the required magnitude increasing rapidly with epicentral distance, and *M*_w_ ≥ 6.5–6.9 for the HF scenario. Notwithstanding the uncertainty associated with the IPE’s discussed previously, the upper range of these magnitudes is consistent with the observation that none of the reported near-field earthquakes with magnitudes *M*_w_ ≤ 5.6 produced a turbidite in Lake Rara, suggesting that the turbidites record earthquakes with magnitudes above the regional background seismicity during the last decades. The rapid increase in required magnitude with epicentral distance is by the fact that the 1980 *M*_w_ 6.5 earthquake at an epicentral distance of ~100 km, and six instrumental *M*_w_ ≥ 6.2 earthquakes in western Nepal and northern India, failed to trigger turbidites in the lake during the last 40 years.

### Implications for seismic activity in western Nepal

The above findings suggest that, on average every 50–100 years since 1505, earthquakes with a minimum magnitude *M*_w_ ≥ 5.3–5.6 have produced turbidites in Lake Rara. These minimum magnitudes are for near-field earthquakes occurring in the immediate vicinity (<15 km) of the lake. Regional earthquakes (≥80 km epicentral distance from the lake), should have magnitudes *M*_w_ > 6.5 in order to trigger turbidites in the lake. Such earthquakes could occur on either the MHT or the WNFS. In both cases, the earthquake slip would contribute to reducing the stored seismic moment in western Nepal, since the WNFS accommodates oblique convergence in the western-central Himalaya through slip partitioning^37,38^. Our data suggest that western Nepal may be as seismically active as central Nepal (Fig. 4) and call for reconsideration of the notion of a seismic gap in western Nepal and northern India. Further testing the seismic-gap hypothesis will require studies involving longer time series and additional lacustrine paleoseismic investigations in this region. Future studies of other Himalayan lakes should allow correlating these events over a broader region. We also note the critical need for IPE calibration in this region in order to better quantify the most likely threshold intensity and magnitude to trigger lake turbidites. By using lake-sediment records as a paleo-seismometer for the first time in the Himalaya, this study complements the record obtained from paleoseismological and historical archives, demonstrating the importance of a holistic and diversified approach in paleoseismology to improve seismic hazard assessment.

## Methods

### Sediment core collection and analysis

In order to reconstruct the past earthquake activity of the Lake Rara area, we collected three short sediment cores with an average length of 40 cm. The coring sites were selected after a preliminary bathymetric survey that complemented existing depth information^40^. The coring sites are located in two different areas at water depths of 168 m and 120 m, respectively (Supplementary Figs. 1, 2). The cores were obtained in October 2014 with an Uwitec gravity corer operated from an inflatable dingy.

Cores were split lengthwise using a Geotek core splitter and described macroscopically. Their physical properties, including γ-ray attenuation density and magnetic susceptibility, were obtained using a Geotek multi-sensor core logger at 2-mm resolution. High-resolution grain-size analysis was performed on core RA14-SC05 with a step of 2.5 mm using a Malvern Mastersizer 3000 after removing organic matter, calcium carbonate and biogenic silica^41^. Grain-size distribution parameters were obtained using Gradistat V8^42^ and were calculated according to ref. ^43^. The relative concentration of major elements was obtained by X-ray fluorescence spectrometry (Itrax XRF core scanner) at 500-μm resolution at the Stockholm University Slamlab (Sweden). Bulk organic geochemistry (TOC, TN) was measured with an elemental analyser (PDZ Europa ANCA-GSL) at the UC Davis Stable Isotope Facility. Before analysis, samples were placed in silver capsules and decarbonated using 1 N sulphurous acid^44^. The amount of sediment was optimized based on preliminary LOI_550_ measurements.

To reconstruct the 3D structure of the split cores we made use of a Siemens Somatom Definition Medical X-ray Computerized Tomography (CT) scanner^45^, which produces CT-slice images composed of voxels (volume elements) with a resolution of 0.15 × 0.15 × 0.60 mm. The 3D reconstruction was obtained by processing the contiguous set of CT slices with VGStudio v2.1. Radio-density values were extracted from the grey levels of the CT-slice images and represented using a colour chart to highlight variations in density within the cores. The CT grey levels correspond to X-ray attenuation, reflecting the proportion of X-rays absorbed or scattered as they pass through each voxel, which is primarily a function of X-ray energy and the density and composition of the material being analysed.

### Age models

A chronology for the Lake Rara sediment record was established on cores RA14-SC05 (site A) and RA14-SC06 (site B) by combining radiocarbon (Supplementary Table 1) and radionuclide (^210^Pb and ^137^Cs; Supplementary Tables 2, 3) dating. Samples for radiocarbon dating were picked outside of the turbidites, as these are considered to be instantaneous deposits.

Radionuclides ^210^Pb and ^137^Cs were measured respectively on 11 and 9 bulk-sediment samples from core RA14-SC05 (Supplementary Fig. 4) by Flett Research Ltd. (Winnipeg, Canada) following the methods of refs. ^46,47^. Radiocarbon ages for cores RA14-SC05 and RA14-SC06 were obtained by dating four and five leaf samples, respectively. After acid-alkali-acid pre-treatment, the samples were converted to graphite following the procedure of ref. ^48^. Isotopic analysis of the graphite targets was performed using accelerator mass spectrometry (AMS) at the University of Tokyo, Japan^49^. All ages were calibrated using the calibration curve for Northern Hemisphere terrestrial ^14^C dates IntCal13^50^.

Age-depth models of cores RA14-SC05 and RA14-SC06 (Supplementary Fig. 2) were produced using Bacon 2.2 software^51^ after removal of the turbidites. The ages indicated in Fig. 2 and Supplementary Fig. 4 correspond to the base of each turbidite

### Modelling shaking intensity

In order to evaluate the potential impact of historical earthquakes on the Lake Rara sediment record, we used custom software built on top of the core Python library of the open-source seismic hazard engine OpenQuake^52^ to compute shaking intensities based on the rupture parameters inferred for these earthquakes and on a set of intensity-prediction equations (IPEs). We evaluated five events: a surface-rupturing earthquake (*M*_W_ ~7.9) on the Tibrikot fault (Western Nepal Fault System; WNFS) between AD 1165 and 1400^37^, the ruptures inferred in ref. ^35^ for the 1833 (*M*_w_ ~ 7.3–7.7) and 1934 (*M*_W_ ~8.1–8.4) earthquakes on the Main Himalayan Thrust (MHT), and the instrumental 1916 Dharchula (*M*_w_ ~7.0–7.2)^28,34^ and 1980 Bajhang (*M*_w_ 6.5)^34^ earthquakes inferred to involve the MHT. The rupture parameters are summarized in Supplementary Table 5. A large number of IPEs is available in the literature, predicting shaking intensity as a function of source and path parameters (mainly magnitude and distance). They differ in the intensity scale (MMI, EMS-98, MSK), magnitude scale (*M*_W_, *M*_S_) and distance metric (epicentral, hypocentral or rupture distance) that is considered, in the geographic area or tectonic region for which they are representative, and in the number and quality of input macroseismic data points. As it is currently not possible to identify a single IPE that is best suited to model shaking intensities for fault ruptures in western Nepal, we applied a mix of five different IPEs: we selected three IPEs^29,31,32^ that were developed for California and other tectonically active regions globally, and performed best in an evaluation for application in the Global Shakemap^53^, supplemented with two IPEs developed specifically for the Himalaya^28,33^. The main parameters of these IPEs are summarized in Supplementary Table 4. Unfortunately, the Himalayan IPEs use a different intensity scale (EMS-98 and MSK, respectively, vs. MMI), but the difference with MMI is probably less than the variability among different IPEs, and one of these studies^28^ made a direct comparison with the two Californian IPEs^29,31^, concluding that there is good agreement in intensity attenuation between the two regions. Using the selected IPEs, we computed maps of mean shaking intensity (Fig. 3a–g) as the arithmetic average of the mean intensity predicted by the different IPEs. We note that, whereas the predicted and observed intensities for the 1934 and 1833 central Nepal earthquakes (Fig. 3e, f) appear to overlap reasonably well (Supplementary Fig. 6), significant discrepancies appear between predicted and observed intensities for the 1980 Bajhang earthquake (Fig. 3a). These discrepancies result both from poorly constrained locations (hypocentre, epicentre, depth) and magnitudes of the modelled events^34^, as well as from the use of IPEs^28,29,31–33^ that are not well calibrated for the region and possibly underestimate intensities (Fig. 3c, d). In particular for the 1916 Dharchula earthquake, the closest recording station used for constraining the source parameters was in Calcutta, at a distance of ~ 10° (>1000 km)^34^. Our intensity map for the 1980 Bajhang earthquake, based on the IPE mix^28,29,31–33^ appears to underestimate the observed near-field intensities^28^ (Fig. 3c, f). In the USGS Shakemap solution, based on one of the IPEs in our mix^29^, it appears that a value of 0.76 has been added to the event magnitude to obtain predicted intensities matching with the observations^28^, explaining why the published Shakemap (https://earthquake.usgs.gov/earthquakes/eventpage/usp0001959#shakemap) shows higher intensities at the site of Lake Rara than our solution. From the IPEs in our selection, it appears that the Himalayan IPE^28^ shows the best match with the observed intensities in the distance range of interest to this study (up to about 150 km), although this is not surprising as ref. ^28^ fitted their IPE to the same observations, which are in EMS-98. Using the IPE from ref. ^28^, the estimated earthquake-recording threshold of Lake Rara would lie between MMI = 5.3–5.7 (Fig. 3).

### Modelling sensitivity to near-field background seismicity

To assess the sensitivity of Lake Rara to moderate-magnitude near-field earthquakes, we estimated the magnitude necessary to cause shaking of intensity MMI ≥ 5.3–5.7 at the lake for different scenarios using the same IPE mix as above. We computed intensities (mean ± 1 standard deviation) for a range of possible magnitudes, and interpolated the magnitude corresponding to MMI = 5.3–5.7 from the obtained magnitude-intensity curves (Supplementary Fig. 8). We considered three different rupture scenarios on the faults that are closest to Lake Rara: one on the MHT directly below Lake Rara (Rara-MHT), and two others on the Dharma (DF) and Humla (HF) Faults, which are both part of the Western Nepal Fault System (WNFS; Fig. 1, Supplementary Fig. 8). Instrumental seismicity in western Nepal mainly occurs around the MHT and is concentrated in two bands north and south of Lake Rara^54^. These scenarios are modelled by selecting a fixed hypocentral location, which is the midpoint of ruptures that increase in size with the considered magnitude. In the Rara-MHT scenario, the hypocentre is directly below Lake Rara at a depth of 26 km^39^. The DF and HF scenarios were modelled at locations corresponding to recent earthquakes, which occurred respectively 2008/12/08 (M_W_ 5.3), and 1980/06/22 (*M*_W_ 5.1). Their source parameters were taken from the Global Centroid Moment Tensor database^55,56^, and we selected the nodal plane that agrees best with the corresponding faults and style of faulting^38,57^. The rupture parameters are summarized in Supplementary Table 6. Because epicentral distance is zero in the Rara-MHT scenario, we decided to leave out IPEs that use epicentral distance, as this may lead to unrealistically high or undefined intensities for this case. In the other scenarios, the distance between the lake and the rupture decreases with increasing magnitude. The magnitude vs. intensity plots obtained for the three scenarios are shown in Supplementary Fig. 8.

## Supplementary information

Supplementary Information

## Data Availability

All the relevant data from this study are available from the corresponding author.
