# Supplementary material for: Potentially large post-1505 AD earthquakes in western Nepal revealed by a lake sediment record
Source: Nat Commun. 2019 May 21;10:2258. doi: 10.1038/s41467-019-10093-4 (PMC6529449; doi:10.1038/s41467-019-10093-4)
Supplement: Supplementary file 1 — Supplementary Information [file 41467_2019_10093_MOESM1_ESM.pdf]

Supplementary Information

Potentially large post-1505 AD earthquakes in western Nepal revealed by a lake sediment record

Z. Ghazoui, S. Bertrand, K. Vanneste, Y. Yokoyama, J. Nomade, A.P. Gajurel, P.A. van der Beek

correspondence to: zakaria.ghazoui@univ-grenoble-alpes.fr

Supplementary Figures

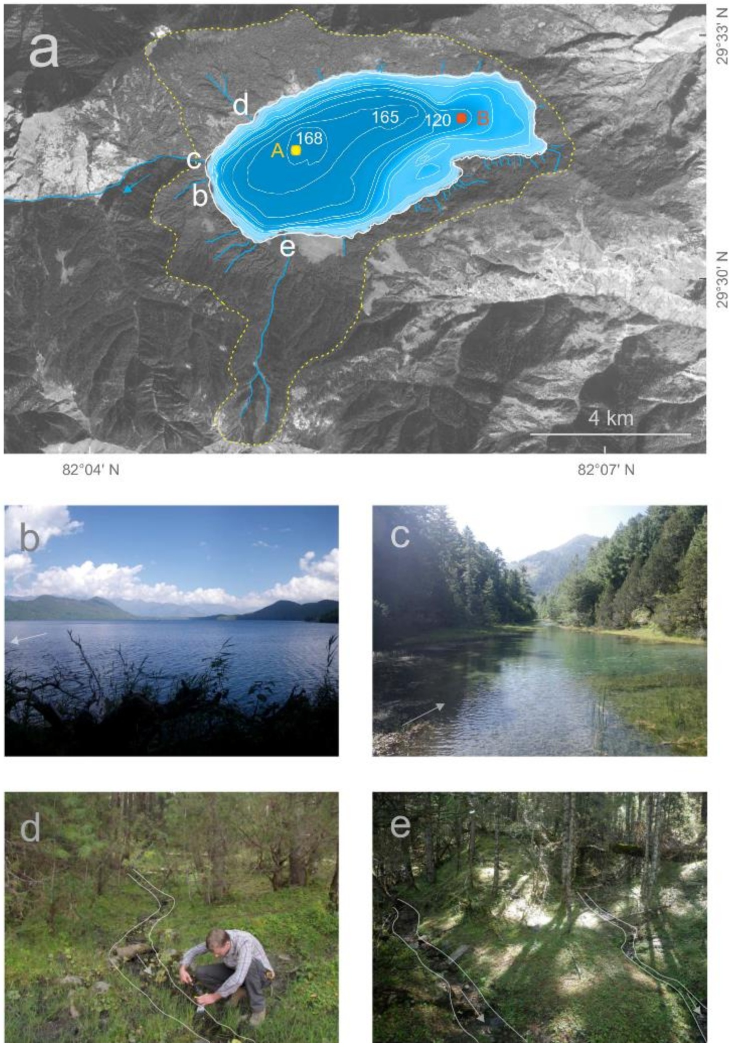

Supplementary Figure 1. Field photos highlighting hydrographic features of the Lake Rara catchment The photos were taken in October 2014, i.e., immediately after the monsoon season (June to early September in 2014), when the rivers are at their highest

level. (**a**) DigitalGlobe image of Lake Rara. The catchment is outlined by the yellow dashed curve. The letters (b, c, d, e) indicate the locations of the corresponding field photos. (**b**) View of the southern bank of the lake, from the east, showing dense forest and gentle slopes around the lake. The arrow indicates the outflow direction. (**c**) Lake outflow channel. The arrow indicates flow direction. (**d**) One of the 37 streams flowing into the lake (north bank), which is of average size and depth. The arrow indicates the flow direction. The cobbles and boulders in the streambed and along the banks of the river originate from moraine deposits intersected by the river; they were probably not transported. (**e**) Two streams on the southeastern bank of the lake, the largest to flow into Lake Rara.

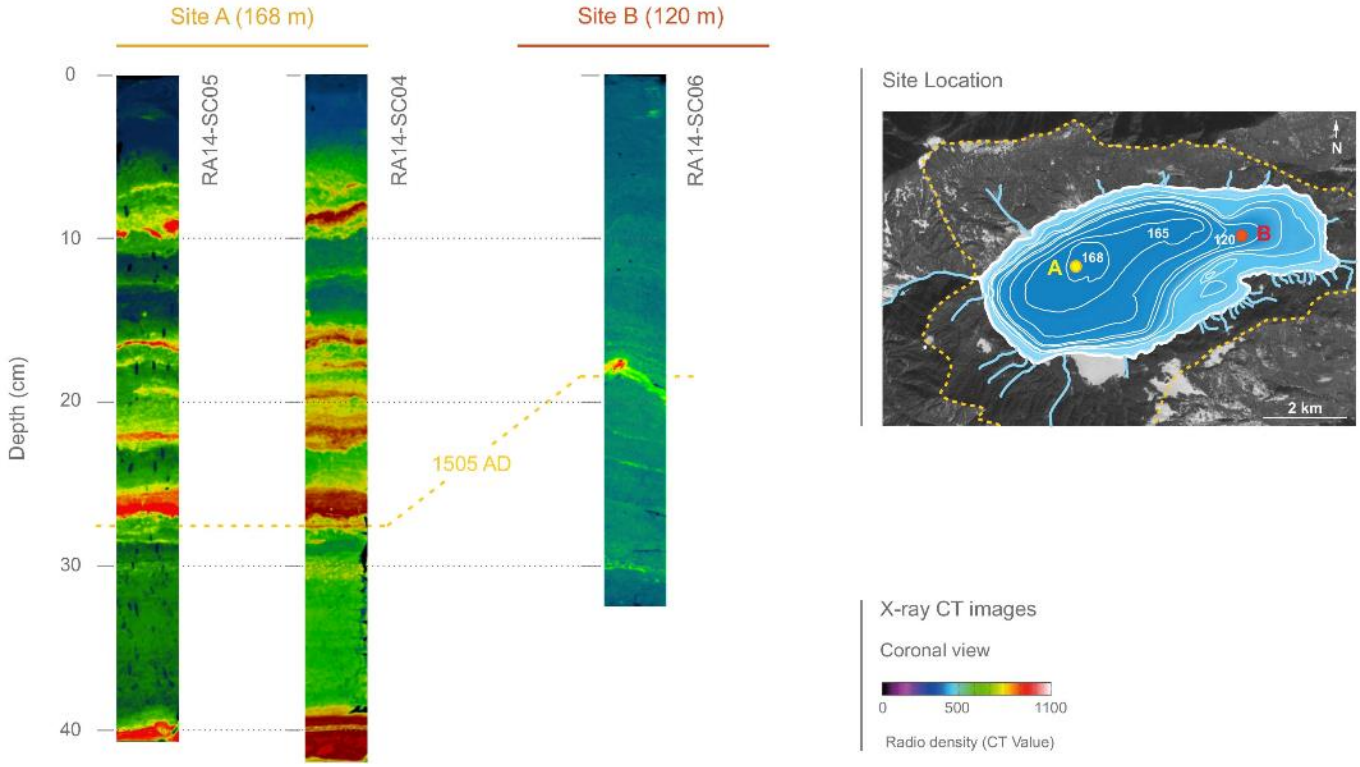

Supplementary Figure 2. Pseudo-colour x-ray computed tomography images of the three sediment cores The images are coronal views of the two cores from site A (168 m water depth) and the core from site B (120 m water depth). The turbidite triggered by the 1505 AD earthquake and correlated between the three cores is indicated.

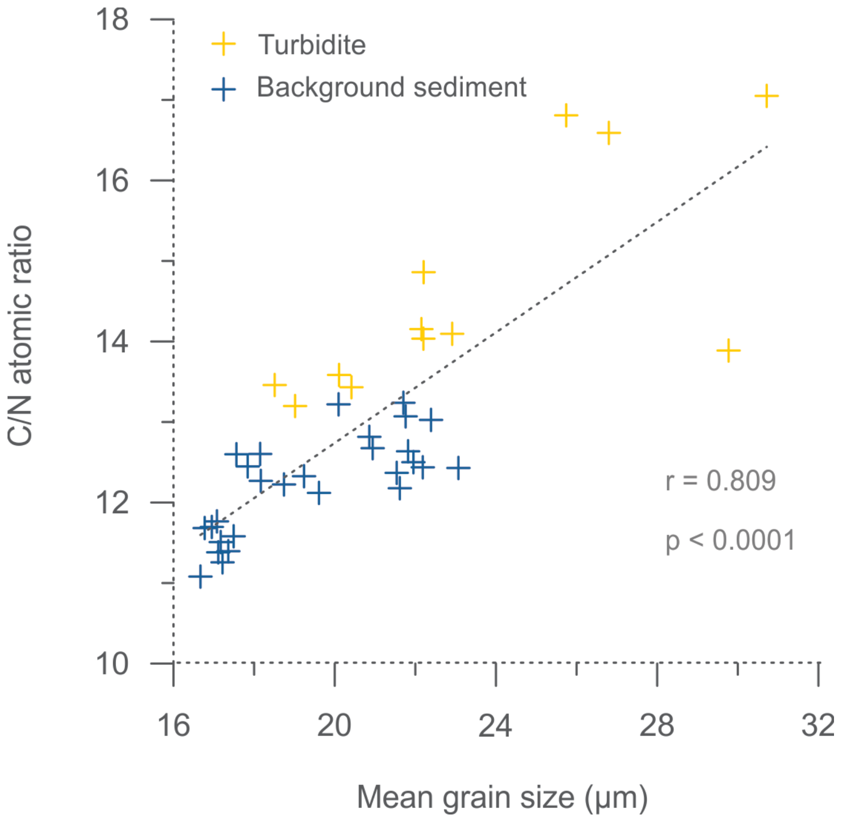

Supplementary Figure 3. Carbon and nitrogen atomic ratio versus mean grain size for samples from sediment core RA14-SC05 (site A) The C/N ratios are significantly positively correlated to grain size ($r = 0.809$, $p < 0.0001$). Note that the C/N values obtained on the turbidites are aligned with the C/N values obtained on the background sediments, implying a strong grain-size control on C/N ratios and a similar source for both turbidite and background sediments.

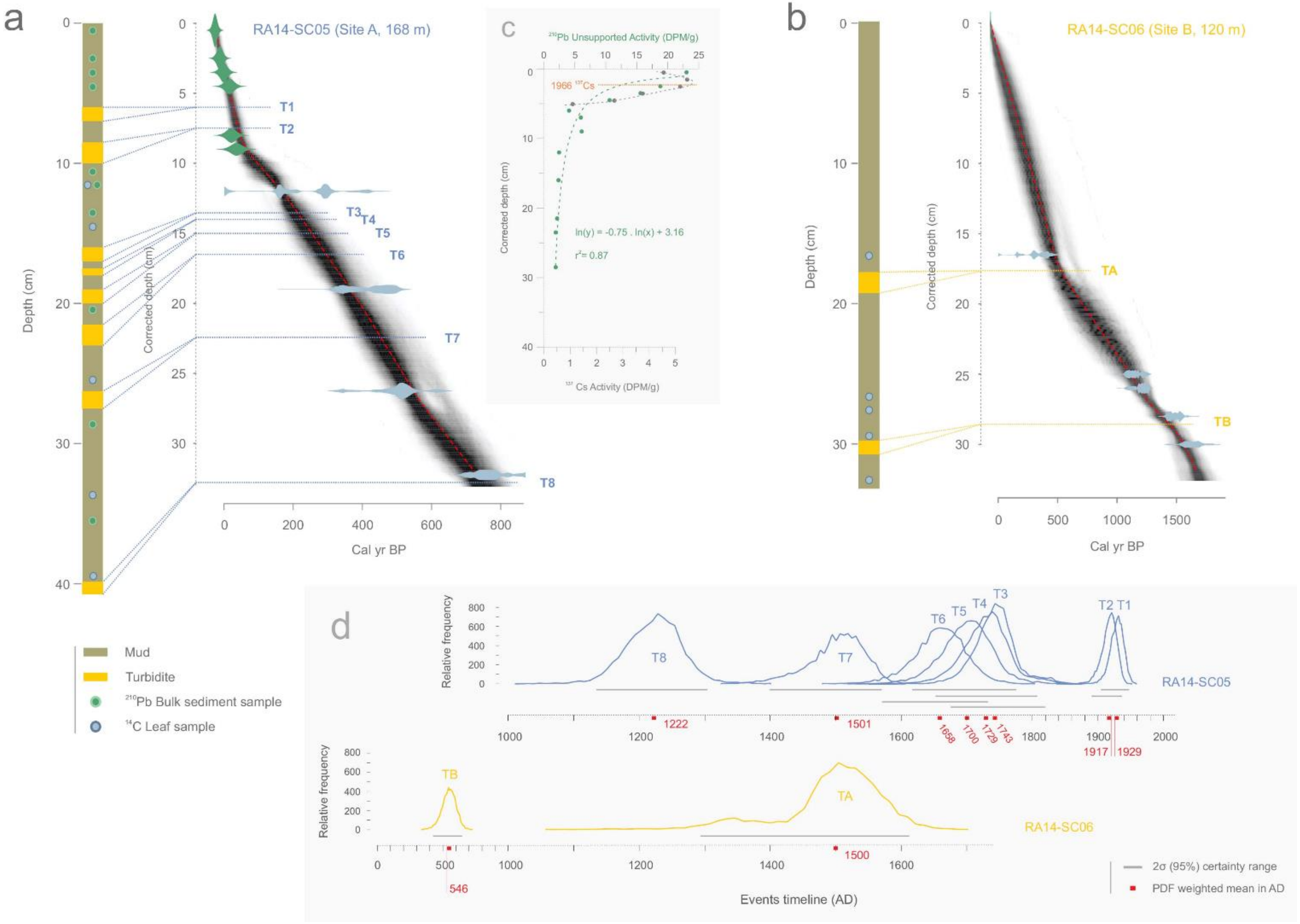

a
RA14-SC05 (Site A, 168 m)
Depth (cm)
Corrected depth (cm)
T1
T2
T3
T4
T5
T6
T7
T8
Cal yr BP
c
^{210}Pb Unsupported Activity (DPM/g)
1966 ^{137}Cs
ln(y) = -0.75 . ln(x) + 3.16
r^2= 0.87
Corrected depth (cm)
^{137}Cs Activity (DPM/g)
b
RA14-SC06 (Site B, 120 m)
Depth (cm)
Corrected depth (cm)
TA
TB
Cal yr BP
Mud
Turbidite
^{210}Pb Bulk sediment sample
^{14}C Leaf sample
d
Relative frequency
T8
T7
T6
T5
T4
T3
T2 T1
RA14-SC05
1222
1501
1658
1700
1729
1743
1917
1929
TB
TA
RA14-SC06
1500
546
Events timeline (AD)
2σ (95%) certainty range
PDF weighted mean in AD

Supplementary Figure 4. Age control Bacon age-depth models[56] for (**a**) core RA14-SC05 (site A) and (**b**) core RA14-SC06 (site B). The corrected depths were calculated by removing instantaneous event deposits (turbidites) from the total depths in order to apply a continuous model in Bacon. The samples analysed for radiocarbon and radionuclides are located on the logs. Grey levels on the age-depth curves represent confidence levels. The red dashed lines represent the overall best fit. Blue dashed lines in (**a**) and yellow dashed lines in (**b**) represent the projection of the turbidites on the age-depth models of core RA14-SC05 and core RA14-SC06, respectively. (**c**) Radionuclide (^{210}Pb and ^{137}Cs) activities as a function of corrected depth for core RA14-SC05. Corrected depth was calculated by removing instantaneous event deposits. ^{210}Pb ages were calculated from the unsupported ^{210}Pb activity using a Constant Rate of Supply (CRS) model. Unsupported ^{210}Pb concentrations were calculated as total – supported (deduced from ^{226}Ra concentrations) ^{210}Pb. The model assumes constant input of ^{210}Pb and a core that is long enough to include all of the measurable atmospheric source ^{210}Pb, i.e. it contains a complete ^{210}Pb inventory. The extrapolated ages were implemented in the Bacon age-depth model. ^{137}Cs concentrations were used as control points for recent deposits since their peak is expected to represent 1963-1966 AD[67]. (**d**) Timeline of the earthquake-triggered turbidites identified in cores RA14-SC05 and RA14-SC06 (in years AD). The Probability Density Function (PDF) of the age of each turbidite is represented in blue for RA14-SC05 and in yellow for RA14-SC06. The grey lines at the bottom of the PDF represent the 2σ (95%) age range for each turbidite. The red squares highlight the weighted mean ages of the events (in years AD).

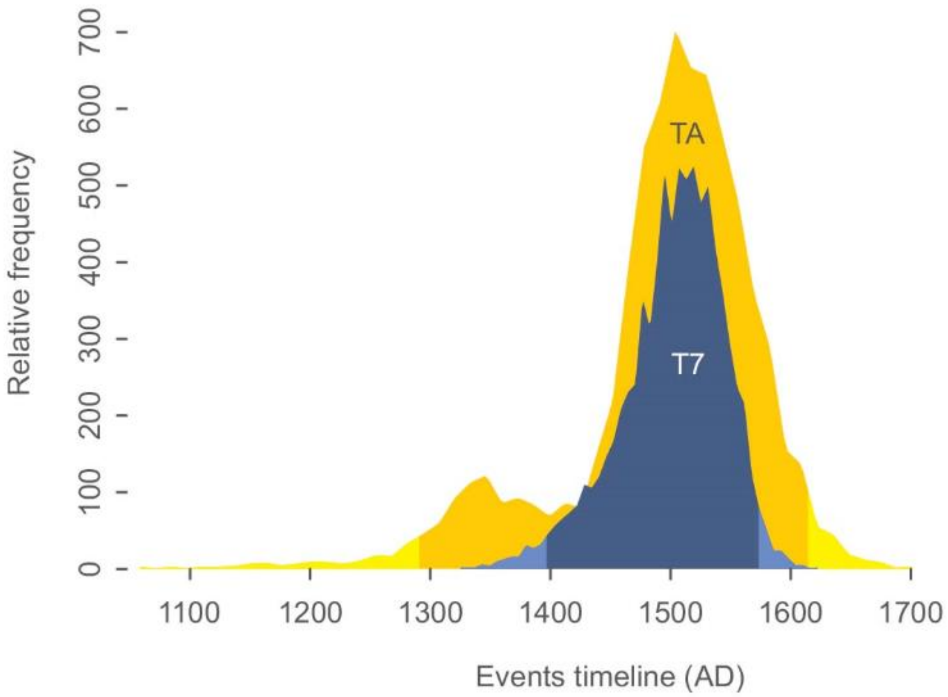

Supplementary Figure 5. Probability Density Functions (PDF) for ages of turbidites T7 (RA14-SC05, site A) and TA (RA14-SC06, site B) Dark blue/yellow represent the 2σ (95%) uncertainty range. Both PDFs peak around 1500 AD, suggesting that the entire body of Lake Rara was affected by a synchronous event. We relate this event to the 1505 AD earthquake.

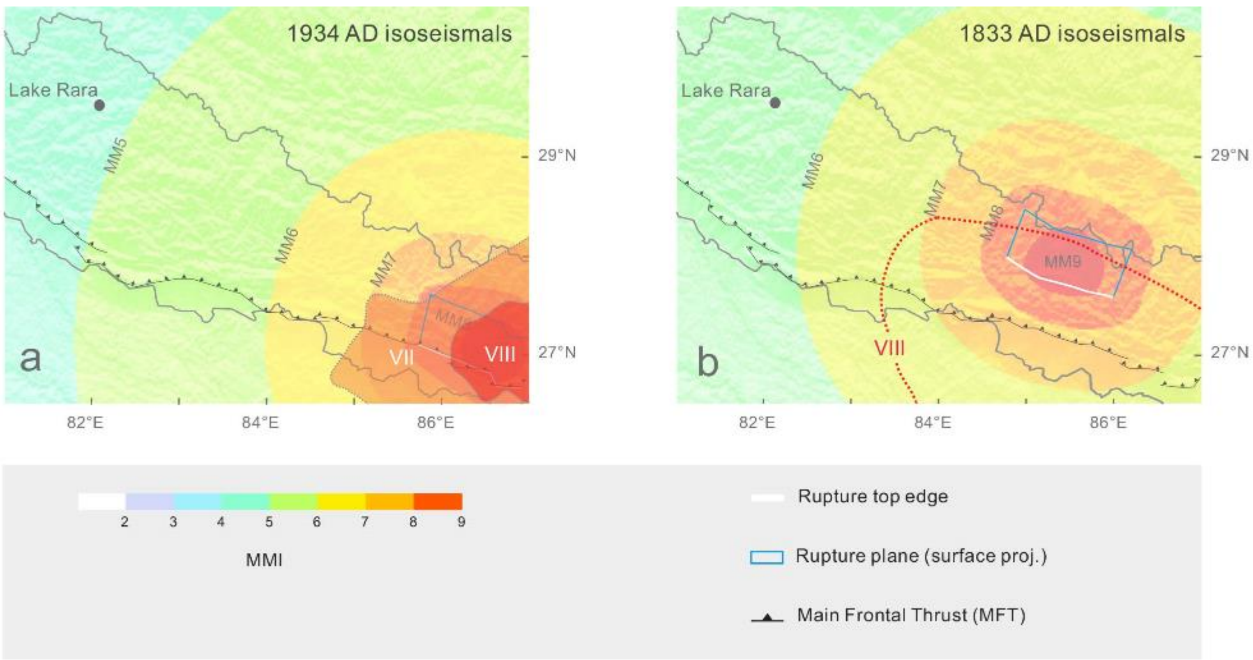

Supplementary Figure 6. Comparison of published and modelled isoseismals for the 1934 AD (modelled M_w 8.4) and 1833 AD (modelled M_w 7.7) earthquakes (a) 1934 AD macroseismic isoseimals (MSK) from ref. 68 (shaded patches with roman numbers) compared to our modelled MMI isoseismals. (**b**) 1833 AD MMI = 8 isoseismal from ref. 69 (dotted contour with roman number) compared to our modelled MMI isoseismals.

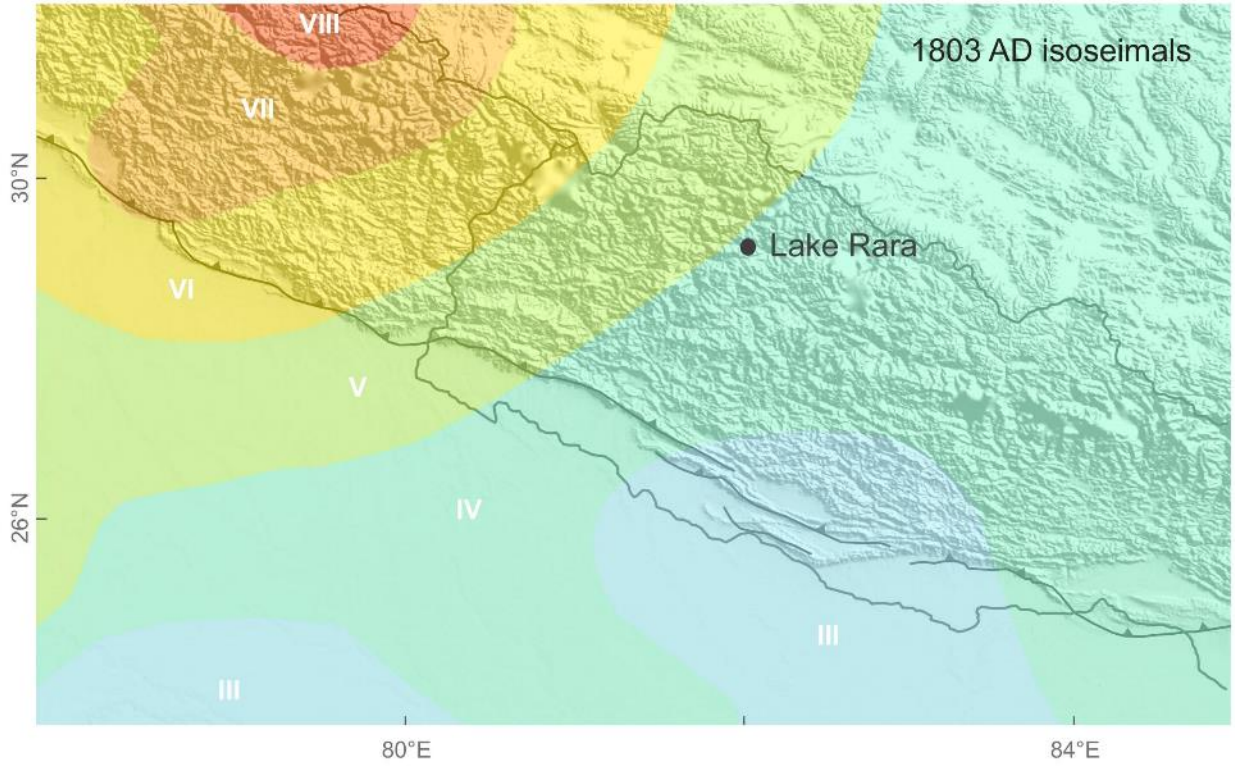

Supplementary Figure 7. Isoseismal map (MSK) of the 1803/09/01 Kumaon earthquake (M_w ~7.3–7.5) The map is redrawn from ref. 29 and based on 33 intensity observations.

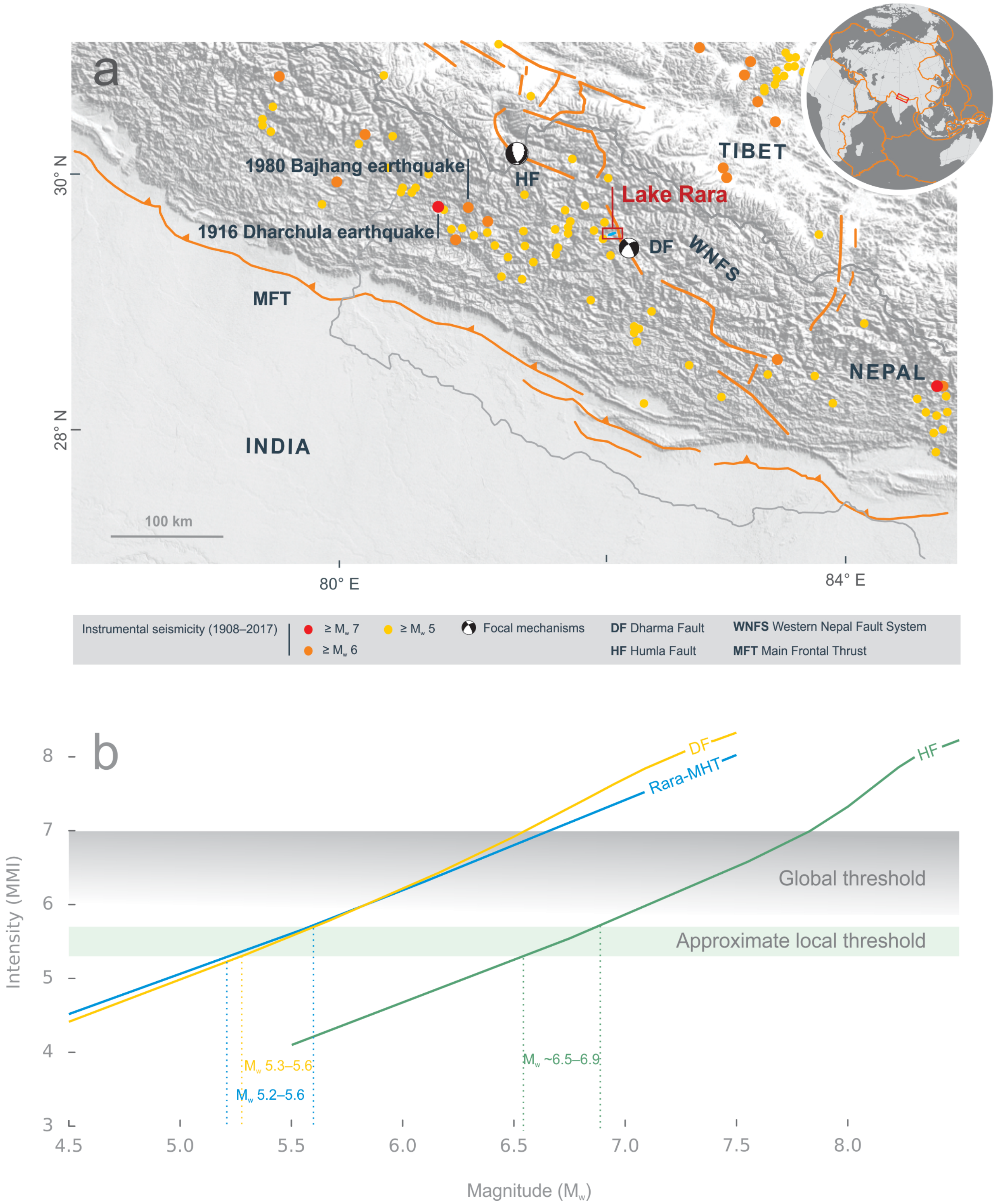

Supplementary Figure 8. **Proximal rupture scenarios and earthquake sensitivity assessment** The first rupture scenario involves a hypothetical earthquake at 26 km depth[39] directly below Lake Rara on the MHT (Rara-MHT), while the other two involve rupture on the Dharma (DF) and Humla (HF) Faults, respectively. (**a**) Map of Western Nepal

illustrating the instrumental seismicity ($M_w \geq 5$) from 1908–2017 (2017 update of ISC-GEM[34]). Representative focal mechanisms from the Global Centroid Catalogue database[60,61] for the Dharma (DF) and Humla (HF) Faults are represented. (**b**) Magnitude versus intensity plot for each rupture scenario, computed using the same IPEs as in Fig. 3 and the source parameters in Supplementary Table 6 (see Methods). The solid curves are the mean magnitude-intensity curves (average of different IPEs) for each scenario. The blue, yellow and green curves correspond to the Rara-MHT, DF and HF scenarios, respectively. The grey shaded rectangle illustrates the earthquake turbidite-triggering threshold (E_QTT) range of MMI 6–7 established at sites globally[21–24,26,27]. The light green rectangle represents the inferred approximate E_QTT range for Lake Rara (MMI ~5.3─5.7).

Supplementary Tables

Supplementary Table 1. AMS ^{14}C ages obtained on sediment cores RA14-SC05 and RA14-SC06.

Core RA14-	*Lab Code*	*Sample depth (cm)*	*Material*	*$\delta^{13}C$ value*	*^{14}C age (yr. $\pm 1\sigma$)*
SC05	YAUT-022721	14-15	Leaf	-17.81	234 ± 35
SC05	YAUT-022723	25-26	Leaf	-25.95	379 ± 47
SC05	YAUT-023023	33-34	Leaf	-18.69	469 ± 51
SC05	YAUT-022724	39-40	Leaf	-27.31	858 ± 44
SC06	YAUT-022725	16-17	Leaf	-22.65	267 ± 48
SC06	YAUT-022726	26-27	Leaf	-19.81	1221 ± 35
SC06	YAUT-022727	27-28	Leaf	-27.45	1258 ± 35
SC06	YAUT-022730	30-31	Leaf	-20.41	1605 ± 36
SC06	YAUT-023013	32-33	Leaf	-31.04	1717 ± 56

Supplementary Table 2. ^{210}Pb data and corresponding CRS ages of samples from sediment core RA14-SC05. The CRS model assumes constant input of ^{210}Pb and a core that is long enough to include all of the measurable atmospheric source ^{210}Pb, i.e., it contains a complete ^{210}Pb inventory. The model assumes that the ^{210}Pb activity of 2.38 DPM/g in the 20-21 cm section (corrected depth 16 cm) corresponds to the background level. The results are plotted in Supplementary Figure 4.

Sample depth (cm)	*Corrected depth (cm)*	*^{210}Pb Total Activity (DPM/g)*	*^{210}Pb Unsupported Activity (DPM/g)*	*CRS age (yr. ± 15%)*
0–1	0.5	23.04	19.70	25.8 ± 3.8
2–3	2.5	18.81	15.48	36.8 ± 5.5
3–4	3.5	15.61	12.28	51.0 ± 7.6
4–5	4.5	10.62	7.29	62.0 ± 9.3
10–11	6	4.08	1.02	79.5 ± 11.9
11–12	7	5.99	2.93	84.0 ± 12.6
13–14	9	6.11	3.05	98.8 ± 14.8
16–17	12	2.48	0.00	
20–21	16	2.38		
28–29	21.5	2.15		
35–36	28.5	1.92		

Supplementary Table 3. ^{137}Cs activities measured on samples from sediment core RA14-SC05. The results are plotted in Supplementary Figure 4.

Lab Code	*Sample depth (cm)*	*^{137}Cs Activity (DPM/g dry wt.)*	*1σ Counting error (DPM/g dry wt.)*
RA14-SC05 (0-1 cm)	0-1	3.70	0.32
RA14-SC05 (1-2 cm)	1-2	4.59	0.62
RA14-SC05 (2-3 cm)	2-3	4.33	0.46
RA14-SC05 (3-4 cm)	3-4	2.91	0.38
RA14-SC05 (4-5 cm)	4-5	1.83	0.37
RA14-SC05 (5-6 cm)	5-6	1.31	0.49
RA14-SC05 (7-8 cm)	7-8	0.44	0.26
RA14-SC05 (9-10 cm)	9-10	0.25	0.20
RA14-SC05 (35-36 cm)	35-36		

Supplementary Table 4. Main parameters of Intensity Prediction Equations used to model macroseismic intensities of different rupture scenarios.

IPE	*Intensity measure*	*Magnitude scale*	*Distance metric*	*Standard deviation*	*Region*
Bakun & Wentworth, 1997[31]	MMI	M_w	Epicentral	Not specified	California
Atkinson & Wald, 2007[32]	MMI	M_w	Rupture	Fixed (0.4)	California
Allen et al., 2012[28]	MMI	M_w	Rupture	Distance-dependent (0.94 at 10 km, 0.76 at 100 km)	Global active crust
Szeliga et al., 2010[30]	EMS-98	M_w	Hypocentral	Not specified	Himalaya
Ghosh & Mahajan, 2013[33]	MSK	M_s	Epicentral	Fixed (~0.246)	NW Himalaya

Supplementary Table 5. Data synthesis on historical/instrumental earthquakes that may have affected Lake Rara and rupture parameters used to compute the intensity maps in Fig. 3 The modelled earthquakes are indicated by a star. Turbidites associated with known historical/instrumental earthquakes are highlighted and the epicentral distance from Lake Rara is indicated.

Earthquake (year AD)	*Catalogue*	*Observed intensity*	*Magnitude*	*Fault*	*Epicentre Latitude (°)*	*Epicentre Longitude (°)*	*Length (km)*	*Mean strike (°)*	*Dip (°)*	*Top depth (km)*	*Bottom depth (km)*	*Epicentral distance (km)*	*Associated turbidite*
1165–1400 *	Historical	-	M_w≈7.9	Tibrikot	-	-	50	302	40	0	20	-	T8
1505 West Nepal	Historical	-	M_s≈8.2	MFT/MHT	≈29.5	≈83	-	-	-	-	-	≈87	T7
1803 Kumaon	Historical	yes	M_w≈7.3–7.5	-	≈31.5	≈79	-	-	-	-	-	≈385	-
1833 Central Nepal*	Historical	yes	M_w≈7.3–7.7	MHT	≈28.3	≈85.5	130	290	10	10	20	≈365	-
1934 Bihar*	Historical / Instrumental	yes	M_w≈8.1–8.4	MFT/MHT	≈26.86	≈86.59	170	283	10	0	10	≈545	-
1916 Dharchula*	Instrumental	yes	M_w≈7.0-7.2	MHT?	≈29.73	≈80.75	-	290	21	20	21	≈132	T2 or T1
1980 Bajhang*	Instrumental	yes	M_w=6.5	MHT?	29.42	80.95	-	290	21	22.3	21	111	-

Supplementary Table 6. Source parameters used for near-field rupture scenarios in Supplementary Fig. 8.

Fault rupture scenario	*Latitude (°)*	*Longitude (°)*	*Depth (km)*	*Strike (°)*	*Dip (°)*	*Rake (°)*
Rara-MHF	29.52	82.09	25	304	26.5	90
DF	29.65	82.01	19.5	328	75	-151
HF	30.10	81.59	15	162	63	-115

Supplementary Discussion

The turbidites were identified using a series of sedimentological and geochemical criteria that included grain size, magnetic susceptibility, Ti concentrations, bulk organic geochemistry and radio-density. From the mean grain-size profiles, the turbidites are identified by their coarse base and a fining-upward sequence of fine sand to very fine silt, in sharp contact with the underlying background mud. Neither the turbidites nor the background sediment contained diatoms. The magnetic susceptibility, the Ti XRF profiles and the C/N ratio exhibit similar patterns as their behaviour is directly related to grain size (Fig. 2; Supplementary Fig. 3). The spikes in magnetic susceptibility are interpreted as recording an input of para- to ferro-magnetic minerals, which are concentrated in the coarse and dense fraction of the sediment. Likewise, it has been shown that Ti concentrations reflect silt content in sediments[62, 63]. The turbidites are most visible on the 3D CT images of the cores, which highlight their dense bases due to their high contents of fine sand to silt, contrasting with the lower radio-densities of the muddy background.

Turbidites within lake sediments can be triggered by various factors such as floods, spontaneous slope failures, or earthquakes. In the main text, we argue why floods or slope failures are unlikely triggering mechanisms for the Lake Rara turbidites.

In the case of Lake Rara the "synchronicity criterion"[23, 64] cannot be directly applied as the lake is composed of a single basin, which means that the two coring sites are not entirely independent. Our best argument for attributing an earthquake origin to Lake Rara turbidites is a temporal correlation with known historical events[22, 23, 65, 66]. We have been able to relate turbidite T7 (1399–1570 AD; Fig. 2; Supplementary Fig. 4) to the great 1505 AD earthquake that ruptured the MFT, but also T8 (1135–1303 AD) to the rupture of the Tibrikot fault segment of the WNFS in 1165–1400 AD[37]. In addition, the T1 or T2 turbidite (1905–1947 AD and 1891–1936 AD, respectively; Fig. 2; Supplementary Fig. 4) can be correlated to the 1916/08/28 Dharchula earthquake (M_w ~7.0–7.2,

29.730°N 80.745°E), which was added to the ISC-GEM catalogue[34] in 2017. By doing so, we have demonstrated that the occurrence of turbidites in Lake Rara is not due to random slope-failure processes but related to seismic activity implying ruptures on both the MFT/MHT and the WNFS.

As stated in the main text, the geomorphologic and hydrographic context renders the potential of flood-triggered turbidites unlikely. The hydrographic system of Lake Rara contains 37 small streams flowing along gentle (<30°) and densely forested slopes (Supplementary Fig. 1). These streams have a maximum length of ~4 km (most are ≪1 km), an average width of 30 cm and a depth of about 15 to 30 cm. The sub-aquatic origin of the turbidites is additionally supported by the C/N signature of the turbidites, which ranges between 13–17, representing a mixture of aquatic (C/N <8) and terrestrial (C/N >20) sources[66]. In contrast, turbidites resulting from a hydrometeorological event would have a terrestrial C/N ratio >20 (ref. 66). The C/N values measured in Lake Rara turbidites are similar to (in fact somewhat more aquatic than) the values measured by ref. 22 in subaqueous mass-wasting deposits in New-Zealand lakes. Although the absolute values depend on grain size (Supplementary Fig. 3), these results suggest that the turbidites originate from the reworking of sediment previously deposited at shallower locations within the lake.

The low hydrodynamic activity in the catchment of Lake Rara is also suggested by the very low inferred sedimentation rates (0.3–0.5 mm/yr at site RA14-SC05 and 0.2–0.3 mm/yr at site RA14-SC06). These are less than half of the sedimentation rates generally observed in lakes used in paleoseismic research (~1 mm/yr or higher[22,27]), resulting in higher slope stability and therefore rendering spontaneous slope failures unlikely. Finally, it is worth noting that sedimentation rates in Lake Rara have not significantly changed during the last millennium (Supplementary Fig. 4a). This observation implies that land-use activities have had little effect on soil erosion and therefore did not affect the rate of sediment remobilisation and the earthquake recording sensitivity of the lake.

Of the eight turbidites recorded in core RA14-SC05 (site A; Fig. 1; Supplementary Figs. 1 and 2), only turbidite T7 (1399–1570 AD), which is attributed to the great 1505 AD earthquake, is also expressed in the sediments of the shallower site 2 (core RA14-SC06; Supplementary Fig. 2). The age of turbidite TA (1285–1606 AD) is statistically indistinguishable from that of turbidite T7 (1399–1570 AD), as demonstrated by the similarity of the Probability Density Functions (PDF) of both turbidites, which both peak around 1500 AD (Supplementary Fig. 5). This synchronicity strongly suggests that turbidites T7 and TA represent the same event, and were likely generated by the same slope failure, which triggered a turbidity current affecting the entire basin of Lake Rara. The reason why the 1505 AD event is the only one recorded at both locations likely reflects the particularly large magnitude of this event, combined with the lower recording sensitivity at site B.

Supplementary References

62. Cuven, S. P. Francus, P. Lamoureux, S. F. Estimation of grain size variability with micro X-ray fluorescence in laminated lacustrine sediments, Cape Bounty, Canadian High Arctic. *J. Paleolimnol.* **44**, 803–817 (2010).
63. Bertrand, S. Hughen, K. A. Sepúlveda, J. Pantoja, S. Geochemistry of surface sediments from the fjords of Northern Chilean Patagonia (44–47°S): Spatial variability and implications for paleoclimate reconstructions. *Geochim. Cosmochim. Acta* **76**, 125–146 (2012).
64. Schnellmann, M. Anselmetti, F. S. Giardini, D. Prehistoric earthquake history revealed by lacustrine slump deposits. *Geology* **30**, 1131–1134 (2002).
65. Bertrand, S. Charlet, F. Chapron, E. Fagel, N. De Batist, M. Reconstruction of the Holocene seismotectonic activity of the Southern Andes from seismites recorded in Lago Icalma, Chile, 39°S. *Palaeogeogr. Palaeoclimatol. Palaeoecol.* **259**, 301–322 (2008).
66. Meyers, P. A. & Teranes, J. L. Sediment organic matter. In: Last, W. M. & Smol, J. P. (eds)

Tracking Environmental Changes Using Lake Sediment, Vol. 2: Physical and Geochemical Methods. Dordrecht: Kluwer Academic, 239–270 (2001).

67. Arnaud, F. Lignier, V. Revel, M. Desmet, M. Beck, C. Flood and earthquake disturbance of ^{210}Pb geochronology (Lake Anterne, NW Alps). *Terra Nova* **14**, 225–232 (2002).
68. Pandey, M. R. Molnar, P. The distribution of intensity of the Bihar-Nepal earthquake of 15 January 1934 and bounds on the extent of the rupture zone. *J. Nepal Geol. Soc.* **5**, 22-44 (1988).
69. Bilham, R. Location and magnitude of the 1833 Nepal earthquake and its relation to the rupture zones of contiguous great Himalayan earthquakes. *Curr. Sci.* **69**, 101–128 (1995).
